# Insights Into the Dynamics and Composition of Biofilm Formed by Environmental Isolate of *Enterobacter cloacae*

**DOI:** 10.3389/fmicb.2022.877060

**Published:** 2022-07-05

**Authors:** Tripti Misra, Meghana Tare, Prabhat Nath Jha

**Affiliations:** Department of Biological Sciences, Birla Institute of Technology and Science, Pilani, India

**Keywords:** biofilms, SERS, *Enterobacter cloacae*, FESEM, medical device

## Abstract

Bacterial biofilms are clinically admissible and illustrate an influential role in infections, particularly those related to the implant of medical devices. The characterization of biofilms is important to understand the etiology of the diseases. *Enterobacter cloacae* are known for causing infections by forming biofilms on various abiotic surfaces, such as medical devices. However, a detailed characterization in terms of morphology and the molecular composition of the formed biofilms by this bacterium is sparse. The present study provides insights into the biofilm formation of *E. cloacae* SBP-8, an environmental isolate, on various surfaces. We performed assays to understand the biofilm-forming capability of the SBP-8 strain and characterized the adhering potential of the bacteria on the surface of different medical devices (foley latex catheter, enteral feeding tube, and glass) at different temperatures. We found that medical devices exhibited strong colonization by *E. cloacae* SBP-8. Using field emission-scanning electron microscopy (FE-SEM) studies, we characterized the biofilms as a function of time. It indicated stronger biofilm formation in terms of cellular density and EPS production on the surfaces. Further, we characterized the biofilm employing surface-enhanced Raman spectroscopy (SERS) and identified the vast heterogenic nature of the biofilm-forming molecules. Interestingly, we also found that this heterogeneity varies from the initial stages of biofilm formation until the maturation and dispersion. Our studies provide insights into biofilm composition over a period of time, which might aid in understanding the biofilm dispersion phases, to enhance the presently available treatment strategies.

## Introduction

The complex and dynamic process of biofilm formation advances through different phases, including initial attachment, micro-colony formation, maturation, and cell dispersion (Van Houdt and Michiels, 2005; Anderson and O’toole, 2008). The bacterial communities engrain themselves in a densely packed matrix comprising extracellular polymeric substances (EPS) to form a complex 3-D architecture of biofilm (Flemming and Wingender, 2010). EPS are primarily composed of polysaccharides, protein, lipids, humic-like substances, extracellular DNA, and water channels (Jefferson, 2004). The produced biofilm matrix provides strength to bacteria against harsh environmental conditions (Flemming et al., 2007). The function of the extracellular matrix within the biofilm is diverse and generally specific to the bacterial species, nutrient(s) availability, and surface properties (George et al., 2005), thereby hinting toward different compositions across diverse microbial species (Wagner et al., 2009). These factors pave the way for diversity and heterogeneity in biofilm formation and hence form the basis for life-threatening biofilm-associated infections (Donlan, 2002). Therefore, it is crucial to identify and characterize comprehensive and molecular composition to know the kinetics of the biofilm formation over the time axis, from the initial phases of attachment to its dispersion. A better understanding of the genetic and molecular mechanisms of biofilm formation may provide strategies for the control of chronic infections and problems related to biofilm formation. Uncovering the roles of EPS matrices in biofilm formation will be beneficial for the design of targeted molecules to control biofilm formation, enhance biocide efficiency, and aid in the development of antifouling agents (Chao and Zhang, 2012).

To characterize the biofilm formation of the bacteria, it is necessary to use a reliable technique that can give us comprehensive information about the biofilm matrix. Primary methods which are widely used to determine biofilm matrix structure and morphology include scanning electron microscopy (SEM), atomic force microscopy (AFM), transmission electron microscopy (TEM), Fourier transform infrared spectroscopy (FTIR), and confocal laser scanning microscopy (CLSM; Ramirez-Mora et al., 2019). These methods have limitations, such as less spatial resolution, non-specificity, being expensive, and being time-consuming (Wagner et al., 2009). Surface-enhanced Raman spectroscopy has recently emerged as an important and reliable technique to understand the heterogeneous and chaotic biofilm matrix. Surface-enhanced Raman spectroscopy (SERS; Sommer et al., 2013) is a non-destructive analytical method that is based on the molecular vibrations derived from the interactions between photons and molecules and provides fingerprint spectra with a high spatial resolution even at low biomass concentration (Hurrell et al., 2009; Ivleva et al., 2010; Sharma and Prakash, 2020). SERS has been used to determine the macro-molecular composition of microbial biofilm matrices and provides insights into the various phases of the progression of biofilms formed by bacteria, fungi, algae, and protozoa (Nivens et al., 1995). A combination of SERS, electron microscopy, and staining assay can reveal detailed information about biofilm.

Clinically, most biofilm-associated infections are implicated by the members of the family *Enterobacteriaceae* (Sommer et al., 2013). Most research on attachment and biofilm formation by the family *Enterobacteriaceae* has focused on *Pseudomonas aeruginosa*, *Klebsiella pneumoniae*, and *Salmonella typhimurium.* However, meager attention has been given to the environmental conditions influencing biofilm formation by *Enterobacter cloacae*, the 10th most isolated nosocomial pathogen. It is actively implicated in causing opportunistic infections, colonizing medical devices, and forming biofilms in various environments (Zhang et al., 2016; Sabir et al., 2017; Ramirez and Giron, 2020). Despite the relevance of *Enterobacter cloacae* as a nosocomial pathogen, the major cause of pathogenicity is still underexplored. Since biofilm formation is considered a major virulence factor imparted by most microorganisms, it becomes increasingly important to understand the biofilm formation by this bacterium. The ability to persist in diverse environments and its virulence make *E. cloacae* a suitable model for this study.

Although the biofilm formation by *E. cloacae* has been reported earlier, the composition of its ECM has not been elucidated. Therefore, the current work aims to gain comprehensive insights into the biofilm formation by *E. cloacae* at varying temperatures of 25 and 37°C and on various surfaces, which are considered the most critical factors influencing biofilm formation (Ochiai et al., 2014). Since environmental isolates can en route to the hospital set up and cause infections, we selected an environmental isolate for evaluating its potential to form biofilm on various surfaces. As our previous study has demonstrated the pathogenic potential of an environmental isolate, *E. cloacae* SBP-8 in animal model organisms (Khan et al., 2020), we selected it as a bacterial model for the study. We characterized the extracellular matrix of biofilm formed by *E. cloacae* employing SERS to understand the highly heterogeneous chemical composition of the biofilm matrix over the time axis of 24–120 (h) at the molecular level. The biofilm formation capability of the given isolate was examined using a simple crystal violet assay, followed by a field emission-scanning electron microscope (FE-SEM) to provide high magnification and high-resolution visualization of the morphology of the biofilm on various surfaces.

## Materials and Methods

### Bacterial Strain, Culture Media, and Conditions

We used *E. cloacae* SBP-8 (Accession No. NAIMCC-B-02025), an environmental isolate obtained from rhizospheric soil (Singh et al., 2017). The culture was grown in Luria Bertani (LB) broth media (HiMedia) at 37°C with agitation (150 rpm) as and when required. The glycerol stocks made with 20% glycerol (SRL) were stored for further use at −70°C.

### Evaluation and Quantification of Biofilm Formation on Various Surfaces

The kinetics of the biofilm formation on various surfaces, such as glass, enteral feeding tubes, and latex catheters, were assayed using the standard crystal violet (CV) assay with brief modifications (discussed in the following section). Enteral feeding tube (Romolene batch no: 171122361, India) and latex catheter (Romolene batch no: G20082297) were used as different surfaces. Culture tube and glass slides (Borosil, India) were used as glass surfaces.

### Biofilm Formation on Medical Devices

Sterile latex catheters (CT) and enteral feeding tubes (EFT) were cut into 0.5 mm thick disks and were aseptically introduced into 5 ml LB broth inoculated with *E. cloacae* SBP-8 (diluted to 1:100). We chose 25 and 37°C for growth and biofilm formation pertaining to the room temperature and the human body temperature, respectively. The tubes were incubated for different periods (24, 48, 72, 96, and 120 h) under static conditions. After respective incubation periods, unattached cells were removed by rinsing the disks of the enteral feeding tube and latex catheter with PBS. The disks were further stained with 1 ml of 0.1% crystal violet. After 30 min of incubation, the crystal violet solution was removed, and the excess stain was rinsed off with a mild wash by PBS buffer. Finally, the biofilm was extracted with 1 ml of 33% glacial acetic acid. After 30 min of incubation, the absorbance of the extracted solution was measured at 570 nm. The resulting absorbance is an indication of the formed biofilm (Philips et al., 2017). The extracted biofilm was diluted two to three times before measurement. The optical densities were measured using a Multiskan GO spectrophotometer (Thermo Scientific, Waltman, MA, United States). All biofilm quantification experiments were done in triplicate (biological replicates).

### Biofilm Formation on Glass Surface

Glass tubes (1 cm diameter) were used to evaluate the biofilm formation. The tube containing 5 ml of LB broth was inoculated with the standardized culture of *E. cloacae* SBP*-*8, which was diluted to 1:100. The tubes were incubated at 25 and 37°C for different time intervals under static conditions. The crystal violet assay to evaluate the biofilm formation was performed as described earlier.

### Observation of Biofilm by Field Emission-Scanning Electron Microscopy

The morphology of biofilm formed on the surfaces of latex catheters, glass slides, and enteral feeding tubes was examined employing FE-SEM using the protocol of Djeribi et al. (2012) with a minor modification. The 0.5 mm thick disks of the medical devices and glass slides were aseptically introduced into the tubes containing LB broth inoculated with *E. cloacae* SBP*-8* with a dilution of 1:100 at 37°C under static conditions. The samples were dehydrated and sputter-coated with gold metal using the Quorum Q150T ES system. The biofilm morphology was observed in a Thermo scientific FEI FE-SEM APREO S SEM system (Netherland) at 20 kV.

### Biofilm Characterization Using Surface-Enhanced Raman Spectroscopy

#### Substrate Preparation

The experiments were conducted using polished crystal quartz optical slides with a thickness of 2 mm and a diameter of 20 mm (TPQ-11, Techinstro, Nagpur, Maharashtra). Before use, the slides were immersed in ethanol: HCl (70:1 v/v) solution overnight, followed by its thorough washing with deionized water. Finally, the slides were heated at 250°C for 4 h in a furnace. The prepared quartz slides were kept strictly in sterilized conditions before use.

#### AuNP Synthesis

The AuNPs are used to enhance the surface for evaluating biofilm formation. The 32-nm AuNPs were synthesized as per the standard Fren’s method (Mahala et al., 2020). First, 1.25 μl of 10^–2^ M HAuCl_4_ and 48.25 ml of water were taken in an Erlenmeyer flask and heated with vigorous stirring, followed by the immediate addition of 625 μl of sodium citrate. We have used AuNPs in our studies owing to their safe and non-toxic nature to the bacteria (Chen et al., 2015; Mahala et al., 2020). The synthesized AuNPs were washed with Millipore water and concentrated by centrifugation at 3,400 *g* for 5 min (Eppendorf centrifuge 5430R, Germany). The supernatant was carefully removed, and the precipitate was utilized for further use.

#### Sample Preparation for Surface-Enhanced Raman Spectroscopy

About 25 ml of LB broth was inoculated with the standardized culture of *E. cloacae* SBP*-8*, which was diluted to 1:100. The bottles were incubated at 37°C for 24, 48, 72, 96, and 120 h under static conditions. The sterilized quartz slides were placed in the inoculated culture for their respective cultivation time. After each cultivation period, the quartz slides were carefully removed and washed three times with PBS solution to remove the unbound cells. About 250 μl of AuNP solution was added in darkness and air-dried before conducting the SERS measurement.

#### Surface-Enhanced Raman Spectroscopy Measurement

The spectra were collected in the range of 400–1,800 cm^–1^, which covered the fingerprint region of most biological materials. SERS spectra were acquired from a LabRAM HR Evolution spectrometer equipped with a He-Ne laser (633 nm, 17 Mw; Japan). The spectrometer was equipped with a grating of 1,800 lines/mm, and the detector was a Peltier air-cooled CCD array detector. Before measurement, the wavenumber calibration of the Raman system was conducted by using a silicon wafer as reference (520 cm^–1^) according to the previous studies. Due to the heterogeneous nature of the biofilm, a total of five spectra from different regions of the slide were scanned. All the procured SERS spectra were pre-processed using baseline correction, normalized to the spectral area in the 400–1,800 cm^–1^ area, and smoothened using Origin software (9.0). Principal component analysis (PCA) was performed using Origin pro (9.0) to reduce the dimensionality of SERS. PCA scores of the first and the second principal components were used to plot 2D charts, based on which differences/similarities of SERS spectra of the biofilm grown for different time durations were analyzed.

##### Statistical Analysis

All experiments were carried out in triplicates and repeated in three independent trial sets. Statistical analysis was performed using Prism 7 (GraphPad Software). Unpaired Student’s *t*-test was performed. Statistical significance: *P ≤ 0.05, **P ≤ 0.01, ***P ≤ 0.001, and ****P < 0.0001, ns = not significant.

## Results

### Evaluation and Dynamics of Biofilm Formation by *Enterobacter cloacae* SBP-8

The ability of *E. cloacae* SBP-8 to form biofilm was evaluated at different surfaces (medical devices such as an enteral catheter, foley catheters, and glass), temperatures (25 and 37°C), and time intervals using CV assay under static conditions. In general, all surfaces used in this study supported biofilm formation favorably at 37°C. The biofilm formation could be observed from 24 h of inoculation, which consistently showed an increasing trend up to 96 h.

**FIGURE 1:**
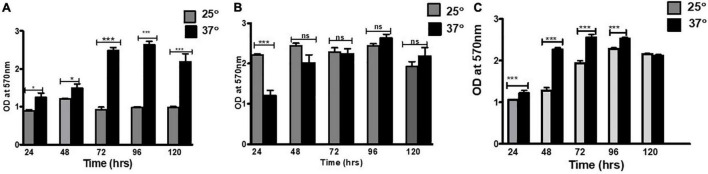
Biofilm formation on various surfaces: **(A)** glass, **(B)** enteral feeding tube, and **(C)** foley latex catheter. To estimate the biofilm production, the crystal violet staining method was used. The graph represents OD_570_ depicting a comparison between 25 and 37°C. An unpaired *t*-test was used to determine significant differences between the two cultivation temperatures. Statistical significance: * ≤ 0.05, *** ≤ 0.001, ns = not significant.

First, the effect of two different temperatures, that is, 25 and 37°C, on *E. Cloacae* SBP-8 was investigated on the glass surface (Figure 1A). Though the biofilm formation was favored at both temperatures, the extent of biofilm formation differed on the glass surface. Figure 1A depicts that the biofilm formation was 2.5-fold higher at 37°C than at 25°C. The initial examination of the CV assay revealed the variation in the adhered biomass of the stained tube (Supplementary Figure 1A). The number of attached bacteria to all the surfaces mentioned earlier has been depicted in Supplementary Figure 2.

There was a consistent increase in the biofilm formation at both temperatures up to 96 h of the cultivation period as represented in OD (optical density). In the CV assay, the mean absorbance ranged from 1.0 to 2.2 at 25°C, whereas it was 1.1–2.1 at 37°C, suggesting 37°C as the favored temperature for biofilm formation.

The biofilm formation by *E. cloacae* SBP-8 on the surface of the enteral feeding tube and foley latex catheter was evident from the initial examination based on CV assay, which revealed the variation in the adhered biomass (Supplementary Figures 1B,C). The kinetic trend of biofilm at 37°C on an enteral feeding tube (Figure 1B) and latex foley catheter (Figure 1C) was consistent with the glass surface. Like the glass surface, biofilm formation on medical devices was enhanced until the 96th hour and declined slightly by the 120th hour. Interestingly, this trend was significantly different at 25°C. The biofilm formation was least in the enteral feeding tube in the initial time points of 24 and 48 h and was constant for later time points of 72–120 h. In contrast to glass and enteral tubes, the latex catheter had higher biofilm formation at 25°C than 37°C up to 72 h. However, on latex foley catheters, higher biofilm formation than observed on glass and enteral tubes was observed at 24 h, indicating a rapid attachment to the surface. However, it exhibited an inconsistent trend of biofilm formation at different time intervals.

### Microscopic Evaluation of Biofilm Formation by *Enterobacter cloacae* SBP-8 Using FE-SEM

**FIGURE 2:**
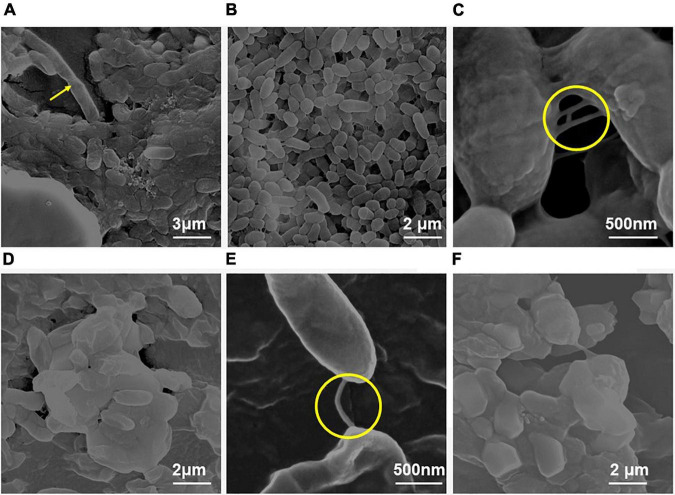
Scanning electron micrographs of the biofilm formed by *E. Cloacae* SBP-8 on an enteral feeding tube at varying time durations from 24 to 96 h. **(A)** Adherence of bacteria onto the surface with a slight production of EPS at 24 h with the presence of few dividing cells (magnification: 24,000×). **(B)** Biofilm formation at 48 h of growth (magnification: 40,000×), with cells engrained in EPS matrix and having smooth morphology. The encircled area shows the presence of nanotubes tubes at 48 h. **(C)** The magnified image of biofilm after 48 h clearly shows thick multilayers of EPS and a nanotube-like structure (encircled area) between the cells (magnification: 1,60,000×). **(D)** At 72 h, clusters of cells were aggregated in a thick layer of EPS (magnification: 40,000×). **(E)** A magnified image of the biofilm after 72 h clearly shows a nanotube joining the two cells in a biofilm (magnification: 1,60,000×). **(F)** Deeply embedded bacterial cells in their EPS matrix at 96 h (magnification: 40,000×).

**FIGURE 3:**
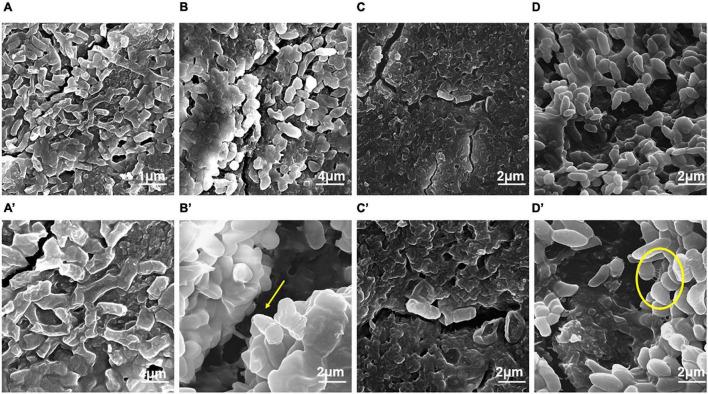
Field emission-scanning electron micrographs of the biofilm by *E. cloacae* SBP-8 on latex catheter at 37°C observed at different time intervals (24–96 h). **(A)** Image of bacterial population in biofilm at 24 h of growth (magnification: 20,000×), **(B)** bacterial cells engrained at 48 h (magnification: 20,000×), **(C)** biofilm at 72 h (magnification: 40,000×), and **(D)** bacterial cells in biofilms at 96 h (magnification: 40,000×). Figures **A’–D’** represent the magnified images of biofilm observed at 24, 48, 72, and 96 h, respectively (magnification: 40,000×). The encircled areas in Figures **A’–D’** show the presence of EPS throughout the period of 24 to 96 h.

The biofilm formed by *E. cloacae* SBP-8 on various surfaces, such as latex catheters, enteral feeding tubes, and glass surfaces, was visualized by FE-SEM. The typical clustering of cells in polysaccharide matrix was observed on latex catheters and enteral feeding tubes (Figures 2 and 3). However, for glass surfaces, we found lesser cellular density and EPS production, leading to weaker biofilms on a flat glass slide than on other medical devices (Supplementary Figure 3).

Field emission-scanning electron microscope analysis showed biofilm on the surface of the enteral feeding tube at all time intervals. It clearly illustrated that the surface was rapidly and promptly colonized by the bacteria with substantial differences throughout 24–96 h (Figure 2). At 24 h, the proliferation and colonization of bacteria appeared on the surface, which was evident by the presence of a few dividing cells (Figure 2A shown with arrow). The cells appeared to be surrounded by a sticky and mucoid layer which is likely to be composed of extracellular polymeric substances (EPS), a characteristic feature of biofilm. At 48 h (Figures 2B,C), an increased number of cells and EPS production were observed. The bacterial colonies were encased in a thick, uniform layer of EPS, and the boundaries between the cells also disappeared significantly. At 72 and 96 h, a cellular multi-layer was observed, as shown in Figures 2D,E, and voids were filled with polysaccharides. With an increase in incubation time, the morphology of the bacterial biofilm changed from smooth to rough. During these periods, the cells were larger than those observed at 24 and 48 h. We also observed the formation of nanotube-like structures between the cells (Figure 2B).

Field emission-scanning electron microscope analysis revealed a similar trend of biofilm formation on the latex catheters as it was seen in the enteral feeding tube. The EPS appeared to be enhancing with time from 24 to 96 h of bacterial growth in biofilm (Figure 3). From 48 h onward, channel-like structures were observed in biofilm. The dark patches shown in Figure 3B’ (with arrow marks) may correspond to the presence of a channel. Like the biofilm formation on enteral tubes, the appearance of nanotubes between the adjacent cells was also observed on the latex catheter surface at 96 h, as shown in Figure 3D’. At 72 h, holes were found in the morphology of the bacterial biofilm, as shown in Figures 3C,C’. Similar structures were also reported by a previous study (Fernández-Barat et al., 2012). The cells of the biofilm were deeply ingrained in the thick multilayers of the EPS and showed different morphology of smooth and rough bacterial surfaces (Figures 3D,D’).

### Study of the Extracellular Composition of Biofilm

**FIGURE 4:**
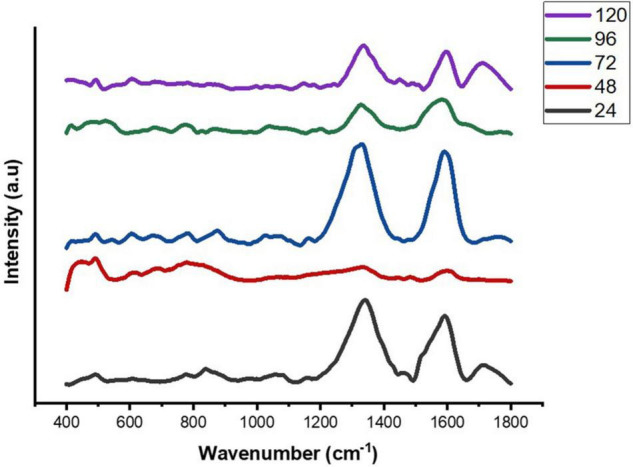
Average SERS spectra of *E. cloacae* SBP-8 biofilms post-cultivation from 24 to 120 h, where (*n* = 5).

We used SERS-based analysis to examine the composition and dynamics of biofilm formation at increasing incubation time (24–120 h) in *E. cloacae* SBP-8. The SERS data showed that the major peaks were prominent in the region marked from 400 to 1,800 cm^–1^ as shown in Figure 4. The SERS spectra depicted various changes over the period and indicated the nature of biofilms being vastly heterogeneous, based on the significant differences between the SERS spectra, Raman peak numbers, positions, intensities, and widths. Owing to the heterogeneity of the formed biofilm, normalized and averaged spectra (*n* = 5) from various regions were used to analyze the variation in spectral intensity. All the procured averaged spectra with respective stack plots were interpreted and are mentioned in Supplementary Figure 4.

**FIGURE 5:**
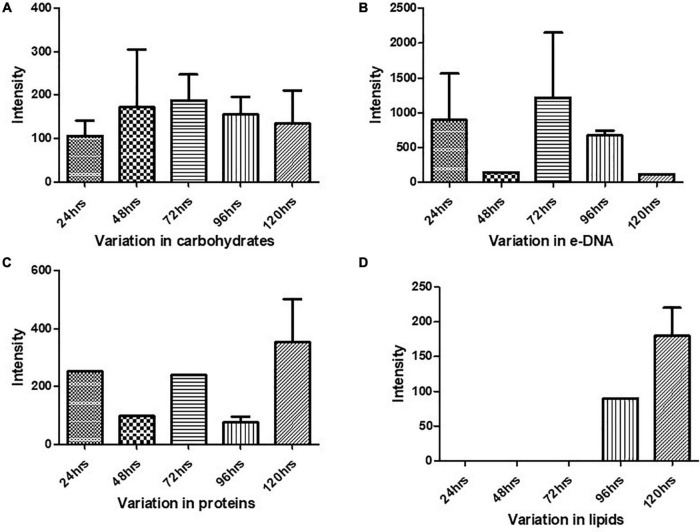
Variation in the biomolecules during the cultivation time period from 24 to 120 h procured through SERS: **(A)** carbohydrates, **(B)** e-DNA, **(C)** proteins, and **(D)** lipids. All the interpreted peaks are exclusive for each of the represented biomolecules.

**FIGURE 6:**
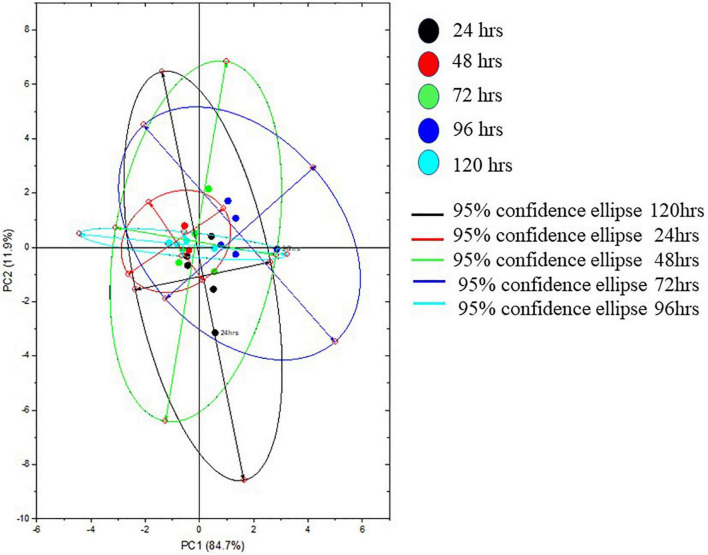
Representative principal component plot from SERS spectra of biofilms formed by *E. cloacae* SBP-8 grown from 24 to 120 h.

The Raman spectra of the formed biofilm demonstrated prominent peaks of the abundant biomolecules present in the biofilm matrix. The details about the peaks and their tentative assignments are given in Table 1. The Raman spectra showed sharp peaks that mostly illustrate the presence of carbohydrates (477cm^–1^), lipids (1,227 and 1,501 cm^–1^), proteins (1,330, 1,341, 1,490, and 1,591 cm^–1^), and e-DNA. On the contrary, less intensity peaks depicting lipids, carbohydrates, and proteins were also seen. Our data also showed a significant presence of amino acids like valine, proline, and tryptophan, as shown in Figure 5, which demonstrated a distinct chemical variation across 24–120 h. These signature peaks represent the abundant biomolecules indicating that carbohydrates, proteins, and e-DNA mark a consistent presence throughout 24–120 h, as shown in Figure 6. Lipids dominated the Raman spectra only for 96–120 h. SERS spectra of the biofilm formed by *E. cloacae* changed with the time duration and showed the heterogeneous presence of the biomolecules. To find the spectral differences among the various time points/duration of the formed biofilm, we used multivariate analysis PCA. The PCA scores of the first and the second principal components were used to plot 2-D charts. The location of the PCA clusters can indicate similarities or differences between the SERS spectra. The ellipses represent an approximate 95% confidence region for each time duration assuming bivariate normality. As shown in Figure 6, PCA clusters of 48 h, 72 h, and 120 h were overlapping, which indicated similar SERS spectral features. However, the data obtained for 24 and 96 h had fewer overlaps with other time points indicative of lesser variation. The variation was seen at 24 h, which is the early time point of the biofilm formation, and at 96 h, which hints toward the ongoing maturation phase of the biofilm.

**TABLE 1:** Summary of the assigned peaks in the SERS spectra.

Biomolecules	Peak assignment (cm^– 1)^	Tentative assignment	References
Carbohydrates	492, 472, 491, 414, 486, 489	Skeletal mode of C-C	Chao and Zhang, 2012
	569, 544	C-O-C glycosidic deformation	Chao and Zhang, 2012
	1041, 1026, 1069, 1058	C-O, C-C stretching	Efeoglu and Culha, 2013
Nucleic acid	776, 785	O-P-O stretching of DNA	Wickramasinghe et al., 2020
	1585	Guanine	Chen et al., 2015
	730	Adenine from flavin	Chao and Zhang, 2012
	1346	Guanine	
Proteins	838	Amide group, deformation vibration	Xie et al., 2013
	1350	Amide II	Henry et al., 2017
	673	C-C stretching tyrosine	Chao and Zhang, 2012
	1028	C-H bending of protein	Ogawa et al., 2020
	869	Single bond stretching vibration of proline and valine	Valle et al., 2008; Goh et al., 2013
	1220–1240	Amide III (arising from coupling of C–N stretching and N–H bonding)	Samek et al., 2008; Chao and Zhang, 2012
	1324	Tyrosine	Xie et al., 2013
	657, 678	C-S stretching and C-C twisting of proline and tyrosine	Efeoglu and Culha, 2013
	852	Ring breathing structure of Tyrosine	Wickramasinghe et al., 2020
	1490	Amide II	
	1596	Amide, Tyrosine	Xie et al., 2013
	1705	Amide I, βsheets	Chen et al., 2015
Lipids	1448, 1452	Deformation vibration of CH_2_ scissoring	Samek et al., 2014
	980	C-C stretching	Samek et al., 2008
	1148	Fatty acids	Ogawa et al., 2020
	1179	C-C stretching vibration	Ogawa et al., 2020

## Discussion

The unsettling incidences of the prevalent biofilm-driven infections and expanding antibiotic resistance have compelled us to understand the nature and complexity of the formed biofilms by *E. cloacae* (Nyenje et al., 2013). We present a comprehensive report that provides insights into the current understanding of the biofilm formation of *E. cloacae* SBP-8 and its chemical composition, which has been sparsely studied.

For the preliminary test of biofilm formation by *E. Cloacae*, we performed a crystal violet assay to evaluate the ability of bacteria to adhere to various surfaces. We observed differential bacterial colonization on medical devices, such as foley latex catheters and enteral feeding tubes. Surface-dependent differential biofilm formation has also been reported in several studies, including higher biofilm formation by *Enterobacter sakazakii* on stainless steel and enteral feeding tubes at 20°C (Kim et al., 2006) and multi-layer of biofilm on silicone catheters by *A. baumanii* (Djeribi et al., 2012).

The significantly higher biofilm formation by *E. cloacae* SBP-8 at 37°C corroborates with the result of *Pseudomonas aeruginosa*, *Klebsiella pneumoniae*, *Vibrio cholerae*, and *Listeria monocytogenes*, which also showed more efficient biofilm formation at 37°C as compared to other temperatures reported previously (Hood and Zottola, 1997; Chavant et al., 2002; Iversen et al., 2004; Uhlich et al., 2014). Further, based on the result of temporal CV assays for biofilm formation, it can be inferred that 24–72 h time can be accounted for the growth and progression of the biofilm. Simultaneously, 96–120 h time point marks a slight decrease in the measured OD, which could be correlated with a shift from the maturation to dispersion phase of the biofilm formation. Our results are congruent with previous reports (Pratt and Kolter, 1998).

Our results from FE-SEM analysis, to understand the morphology, indicate stronger biofilm formation in terms of cellular density and EPS production on the surface of medical devices than observed on the glass surface. It suggests that these surfaces (latex catheters and enteral feeding tubes) could serve as a potent source of infection of *E. cloacae* SBP-8. Laboratory studies have shown that the complex process of biofilm formation was elucidated by visualizing biofilms after different cultivation times. We observed changes in bacterial morphology from smooth to rough with the increasing cultivation time. It was also seen that the drastic changes in morphology were more prominent at 48 h in both medical devices. EPS was also prevalent on medical devices, which is considered a hallmark of biofilm. EPS serve as an essential component of the cell to adhere to the varying surfaces of an implant device. Several reports on *Staphylococcus aureus* and *Proteus mirabilis* have shown the presence of EPS at 48 h of cultivation (Takahashi et al., 2015). It was also demonstrated in urinary catheters infected with the mixed cultures of *Acinetobacter baumanii* and *Pseudomonas aeruginosa*, which formed interconnected multilayers of EPS (Djeribi et al., 2012). In some of the samples, we observed nanotubes with a diameter of > 100 nm, as shown in Figures 2B and 3D’, formed between the neighboring cells of a biofilm at the surface of the medical devices at both 48 and 96 h. Similar to our observations, the appearance of tube-like projections of ca. 100 nm between the cells and the substrate was also observed in earlier reports (Dubey et al., 2016; Baidya et al., 2018). Such bacterial nanotubes are membranous intercellular bridges connecting neighboring cells situated in proximity. The nanotube formation can be correlated with the initial stages of a bacterial biofilm formation; it helps in the establishment of a biofilm and provides the foundation for unrestrained intercellular molecular flow between its inhabitants (Baidya et al., 2018). Nanotubes are also contemplated to substantiate cell–cell connections to contest nutritional stress (Bridier et al., 2015). Our results are congruent with the reports mentioned earlier. The dark patches shown in Figures 3B’ and C’ correspond to the presence of channels that help in the dissemination of nutrients, water, and enzymes in the bacterial biofilm (Bogino et al., 2013; Asahi et al., 2015). Several other studies also showed that channel-like structures are commonly seen in the biofilms formed by *P. mirabilis* on stainless steel at 96 h of cultivation (Fernández-Delgado et al., 2015).

To understand the composition of the biofilm matrix and its dynamics, we employed SERS, a non-invasive method that provides an overall estimation of the chemical components present in a biological substance and helps unravel their relative abundance (Chao and Zhang, 2012). The Raman spectra obtained by SERS provide information on the overall chemical composition, including total carbohydrates, proteins, lipids, and e-DNA. The intensities of the individual components help to elucidate the chemical variations occurring during the various phases of the progression of biofilm (Wagner et al., 2009; Efeoglu and Culha, 2013; Samek et al., 2014; Henry et al., 2017). SERS is extensively employed for single-cell, bacterial colony, and biofilm formation studies (Chao and Zhang, 2012; Efeoglu and Culha, 2013; Chen et al., 2015; Henry et al., 2017). In the current study, we used SERS to detect the biochemical differences in the biofilm formed by *E. cloacae* SBP-8 at various phases, which have been sparsely studied. The SERS mainly analyzed the composition of the EPS matrix, which includes polysaccharides, proteins, nucleic acids (DNA-RNA), and humic-like substances (Chen et al., 2015; Ramirez-Mora et al., 2019). Based on different peaks observed at various time intervals, the dynamics of the constituents of the biofilm matrix are discussed in the following sections.

### Carbohydrates

Our results showed a slow and consistent increase in carbohydrates from 24 to 72 h Figure 5A, which indicates the gradual shift from initial adhesion to micro-colony formation in the biofilm matrix (Kives et al., 2006; Chao and Zhang, 2012). The Raman peaks assigned for carbohydrates are 408–423, 479–495, 565–582, 1,058, 1,055, 873, 1,041, and 1,026 cm^–1^ as mentioned in Table 1. It can be observed from Figure 5A that at 96–120 h, there was a decrease in the carbohydrate content in the matrix, which could be mostly due to the slower metabolic activity of the microorganisms when they encounter stress conditions (Oliver, 2000). Overall, our findings align with earlier studies, which reported that members of the family *Enterobacteriaceae*, such as *K. pneumoniae, E. coli*, and *P. aeruginosa*, predominated polysaccharides in their associated biofilm matrix (Kusić et al., 2015). The consistent presence of exopolysaccharides during the tested period indicates the vital function of exopolysaccharides as a scaffold for lipids, proteins, and e-DNA to adhere to each other (Kostakioti et al., 2013).

### Nucleic Acids

Extracellular DNA (e-DNA) is essential for biofilm development and maturation in many bacteria (Tang et al., 2013; Nguyen and Burrows, 2014). The Raman peak at 730, 1,341, 1,585, 785, 730, 826, and 1,346 cm^–1^ are assigned to DNA backbone (Çulha et al., 2008; Kahraman et al., 2009; Ivleva et al., 2010; Efeoglu and Culha, 2013; Xie et al., 2013; Chen et al., 2015). The Raman bands at 730 cm^–1^ are considered as the nucleic acid marker, which was also found in our study at 72 h (Efeoglu and Culha, 2013). At 24 h (Figure 5B), there was a significant increase in the presence of e-DNA, which can be accounted for the initial adhesion and attachment phase to the surface. e-DNA is proposed to provide structural integrity and stabilize the biofilm matrix, as other EPS components are not initially produced in higher amounts (Gonzalez et al., 2014; Okshevsky and Meyer, 2015). Studies on *Staphylococcus aureus* and *Listeria monocytogenes* suggested that e-DNA is a key entity during the transition from initial to early biofilm formation. A recent study has proven that e-DNA is produced more during the switch from the planktonic state toward the biofilm state in the static condition (Alhede et al., 2020). However, a radically high amount of e-DNA was also found at 72 h (Figure 5B), which marks the maturation phase of the biofilm matrix. This increased amount of e-DNA might have resulted from the lysis of a bacterial subpopulation in response to the quorum-sensing system (Allesen-Holm et al., 2006). The presence of e-DNA gives a strong indication that they are a prominent and strong component of the mature biofilm rather than the initially adhered biofilms (Chao and Zhang, 2012).

### Proteins

Proteins form a crucially extensive component of the biofilm matrix by providing structure and stability to the biofilm. The Raman peaks at 838, 1,350, 673, 1,028, 678, 826, 869, 1,200, 1,327, 657, 852, 1,000, 1,243, 1,490, 1,596, and 1,705 cm^–1^ correspond to proteins (Table 1). The spectra show a significant increase in the protein content at 24 h Figure 5C, which could be explained due to the increased density of microorganisms in the initial phase of the biofilm formation (Chao and Zhang, 2012). Apart from the above-mentioned peaks, the peaks at 838 (amide group I), 1,350 (amide group III), 1,200 (Amide III), 1,243 (Amide III), 1,490 (Amide III), and 1,790 cm-1 (Amide I) were steady in their appearance throughout the period from 24 to 120 h, which indicates the protein secretion as an ongoing process contributing to the biofilm matrix and architecture (Keleştemur and Çulha, 2017).

In addition to proteins, peaks corresponding to certain amino acids were also observed at different time intervals. From 72 to 120 h (Figure 5C), there has been a predomination of amino acid tyrosine (673, 826, 1,327, 657, 852, and 1,596 cm^–1^) in the biofilm matrix. This amino acid is usually secreted in the stationary phase (Kolodkin-Gal et al., 2010). It has already been established in previous studies that tyrosine inhibits biofilm formation and triggers biofilm disassembly (Yu et al., 2016). The highest peak of tyrosine was observed at 120 h, which can be correlated to the dispersion phase of the formed biofilm. Tyrosine is released during the depletion of nutrients as a stress signaling molecule that aids in the dispersion of the old biofilms (Cava et al., 2011). Other amino acids like proline and valine (869 cm^–1^) were also secreted at 96 h. Valine is known to be produced in the biofilm by the members of the family *Enterobacteriaceae*, which primarily includes *E. coli, K. pneumoniae, E. cloacae, Salmonella serovar Enteritidis*, and *P. aeruginosa* (Goh et al., 2013). The secretion of valine is mostly a metabolic adaptation that occurs within the mature biofilm and has been reported in the work done by Valle et al. (2008).

### Lipids

Previous studies have documented the occurrence of signature Raman peaks for lipids generally noticeable in the following regions: 1,500–1,400, 1,300–1,250, and 1,200–1,050 cm^–1^ (Czamara et al., 2015; Kurouski et al., 2015; Keleştemur et al., 2018). Our results also showed peaks pertaining to lipids at 980, 1,452, 1,069, 1,148, 1,179, and 1,448 cm^–1^. SERS spectra indicate that the lipids became prominent in the later stages of the formed biofilm (Figure 5D), that is, 96–120 h, hinting toward the maturation phase of the biofilm. Our inference is consistent with the earlier reports that indicated the presence of lipids during the maturation phase of the biofilms in Gram-negative bacteria (Keleştemur et al., 2018). It has also been shown that lipid content increases in response to the nutrient-deprived condition as an adaption and survival strategy for the bacteria (Kusić et al., 2015).

Overall, our data showed differences in the spectra from a time period of 24–120 h. The PCA score plot of Raman spectra clearly indicated different biochemical compositions. The main spectral differences were noticed in the spectral bands around 400–700, 700–1,000, 1,005–1,283, 1,253–1,548, and 1,545–1,799 cm^–1^. Taken together, the SERS data showed an abundance of major biomolecules in the biofilms formed by *E. cloacae* SBP-8 where carbohydrates played a consistent role throughout the biofilm formation and served as a platform for other biomolecules to adhere to each other. The e-DNA also marked a consistent presence from 24 to 72 h and declined from 96 to 120 h, which shows a remarkable contribution in the progression from the attachment to the maturation phase of the biofilm. The data show lipid production only from 96 to 120 h, which can be accounted for nutritional deprivation encountered during the maturation of biofilms. Our data identified signature molecules pertaining to all the major biomolecules from a time duration of 24–120 h, which are in alignment with other *Enterobacteriaceae* members (Chao and Zhang, 2012; Feng et al., 2015; Henry et al., 2017; Sharma and Prakash, 2020). However, unlike other reports, we also found an abundance of e-DNA at higher concentrations in our data, which probably helps the biofilm to progress radically toward the maturation phase. Apart from this finding, our data also highlight the presence of amino acid tyrosine which was produced at later time points, highlighting toward the dispersion phase. The SERS results well correlated with crystal violet assay and SEM analysis in demonstrating their feasibility for quantifying biofilm morphological changes and EPS production, and determining chemical composition simultaneously.

Our report is likely to be the first one to provide comprehensive insights into the biofilm matrix of *E. cloacae*, a member of the ESKAPE pathogens (Enterococcus *faecium*, *Staphylococcus aureus*, *Klebsiella pneumoniae*, *Acinetobacter baumanii*, *Pseudomonas aeruginosa*, and *Enterobacter species*), which are the leading causes of nosocomial infections throughout the world. As discussed, biofilm development progresses through major stages, including attachment, micro-colony formation, maturation, and dispersion. Although previous reports have only been able to depict biofilm formation from attachment to micro-colony or maturation phase using SERS (Chao and Zhang, 2012; Feng et al., 2015; Keleştemur et al., 2018), the current study depicted all the stages of biofilm progression with distinct phase switching from attachment to early accumulation and then to maturation, followed by dispersion based on chemical and physiological assessments. Studying biofilm for longer cultivation times enhances our understanding of the dynamics and phase switching of the biofilms. In our study, monitoring of biofilms gave us a vivid idea of the dispersion phase, which can be induced by environmental stresses (e.g., starvation and accumulation of toxic wastes) and regulated by signaling molecules. The identification of a specific molecule synthesized at the dispersion stage would provide further evidence for identifying this important stage.

## Conclusion

In the present study, we examined the ability of *E. cloacae* SBP-8 to form biofilm on the surface of medical devices and conducted a compositional analysis to determine the dynamics of biofilm formation at various time intervals. The *E. cloacae* SBP-8 forms biofilm optimally at 37°C. The stronger biofilm formation by *E. cloacae* SBP-8 on medical devices indicates the possibility of an environmental isolate as a source of nosocomial infection. The observation of the nanotube formation between the neighboring cells suggests that the *E. cloacae* in biofilms may communicate with one another through such nanotubes. The present work employed the SERS technique for understanding in detail the macro-molecular composition and dynamics of biofilm. The SERS data revealed the differential level of carbohydrates, proteins, amino acids, lipids, and e DNA at different phases of biofilm. The information related to major macromolecules during the maturation and dispersion phase can be utilized to develop anti-biofilm strategies.

## Data Availability Statement

The original contributions presented in the study are included in the article/Supplementary Material, further inquiries can be directed to the corresponding authors.

## Author Contributions

TM and PJ designed the work, analyzed the work, and helped in the manuscript preparation. TM carried out all the experiments. MT had helped and contributed to the microscopy analysis and editing of the manuscript. All authors read and approved the manuscript.

## Conflict of Interest

The authors declare that the research was conducted in the absence of any commercial or financial relationships that could be construed as a potential conflict of interest.

## Publisher’s Note

All claims expressed in this article are solely those of the authors and do not necessarily represent those of their affiliated organizations, or those of the publisher, the editors and the reviewers. Any product that may be evaluated in this article, or claim that may be made by its manufacturer, is not guaranteed or endorsed by the publisher.
