# Supplementary material for: Insights Into the Dynamics and Composition of Biofilm Formed by Environmental Isolate of Enterobacter cloacae
Source: Front Microbiol. 2022 Jul 5;13:877060. doi: 10.3389/fmicb.2022.877060 (PMC9294512; doi:10.3389/fmicb.2022.877060)
Supplement: Supplementary file 1 [file Data_Sheet_1.docx]

**Insights into the dynamics and composition of biofilm formed by environmental isolate of *Enterobacter cloacae***

Tripti Misra, Meghna Tare, Prabhat Nath Jha

Department of Biological Sciences,

*Birla Institute of Technology and Science, Pilani-333031, Rajasthan, India*

**Supplementary Figures:**

**
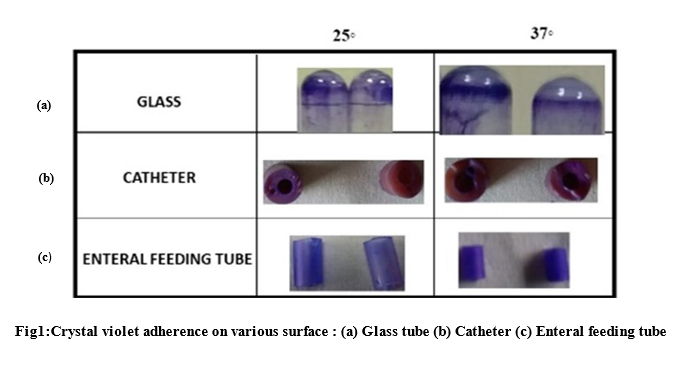
**

Figure S1. Crystal violet assay showing basic adherence on various surface (a) glass (b)foley latex catheter (c) enteral feeding tube at varying temperature 25°C and 37°C.


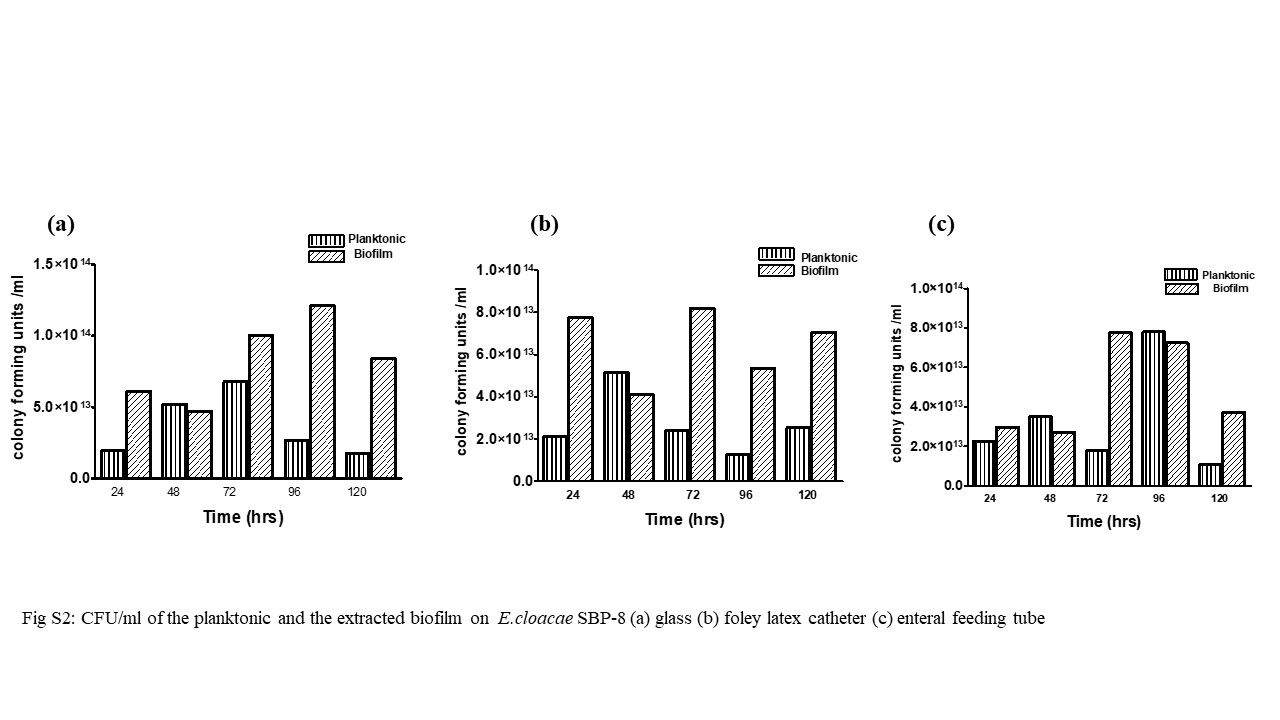
Figure S2: CFU/ml of the planktonic and the extracted biofilm on *E. cloacae* SBP-8 on (a) glass (b) foley latex catheter (c) enteral feeding tube


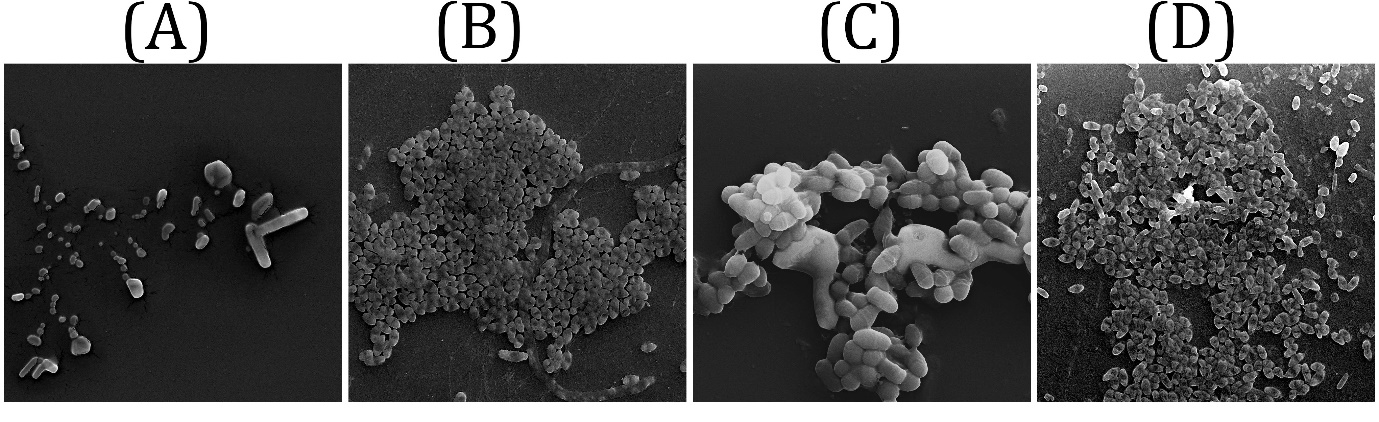


Figure S3: Biofilm formation on the glass surface (A) Slight adhesion of *E. cloacae* SBP-8 at 24 hrs (10,000X). (B) Microcolony formation on the surface at 48 hrs (C) and (D) (10,000X). Microcolony formation with slight production of EPS at 72 and 96 hrs respectively (10,000X).


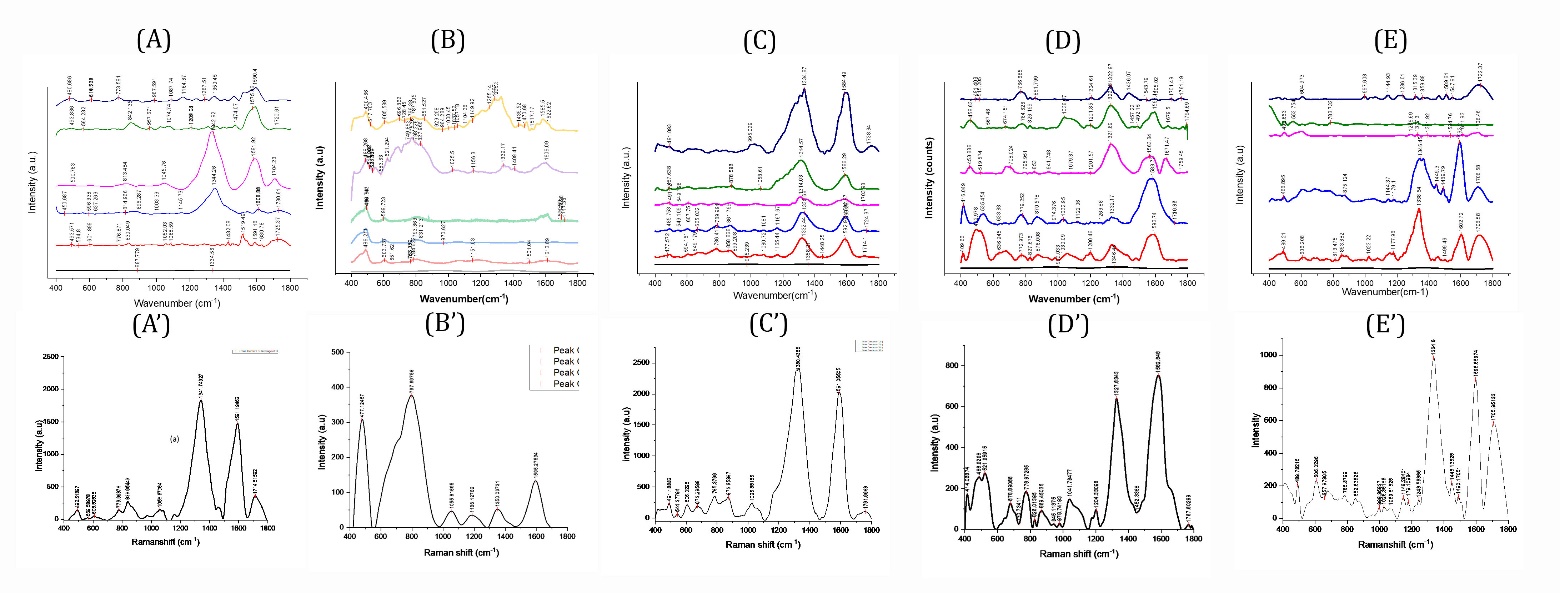


Figure S4: The SERS spectra of *Enterobacter cloacae* SBP-8 biofilms from 24-120(hrs). (A-E) in the upper panel shows the stack plot of the respective time periods where (n=5). (A’-E’) shows the averaged spectra of the respective time periods 24-120 (hrs).
